# Sablefish (*Anoplopoma fimbria*) chromosome-level genome assembly

**DOI:** 10.1093/g3journal/jkad089

**Published:** 2023-04-25

**Authors:** Anne-Marie Flores, Kris A Christensen, Briony Campbell, Ben F Koop, John S Taylor

**Affiliations:** Department of Biology, University of Victoria, 3800 Finnerty Road, Victoria, BC, V8W 2Y2, Canada; Department of Biology, University of Victoria, 3800 Finnerty Road, Victoria, BC, V8W 2Y2, Canada; Golden Eagle Sablefish, 335 Walkers Hook Road, Salt Spring Island, BC, V8K 1N7, Canada; Department of Biology, University of Victoria, 3800 Finnerty Road, Victoria, BC, V8W 2Y2, Canada; Department of Biology, University of Victoria, 3800 Finnerty Road, Victoria, BC, V8W 2Y2, Canada

**Keywords:** teleostei, cottioidei, sablefish, *Anoplopoma fimbria*, genome assembly

## Abstract

Sablefish (*Anoplopoma fimbria*) are in the suborder Cottioidei, which also includes stickleback and lumpfish. This species inhabits coastal regions of the northeastern and northwestern Pacific Ocean from California to Japan. A commercial fishery for sablefish began to flourish in the 1960s, though a downward trend in stock biomass and landings has been observed since 2010. Aquaculture protocols have been developed for sablefish; eggs and sperm from wild-caught and hatchery-reared captive broodstock are used to generate offspring that reach market size in about two years. Parentage analyses show that survival in aquaculture varies among families. Growth rate and disease resistance also vary among individuals and cohorts, but the extent to which genetics and the environment contribute to this variation is unclear. The sablefish genome assembly reported here will form the foundation for SNP-based surveys designed to detect genetic markers associated with survival, growth rate, and pathogen resistance. Beyond its contribution to sablefish domestication, the sablefish genome can be a resource for the management of the wild sablefish fishery. The assembly generated in this study had a length of 653 Mbp, a scaffold N50 of 26.74 Mbp, a contig N50 of 2.57 Mbp, and contained more than 98% of the 3640 Actinopterygii core genes. We placed 620.9 Mbp (95% of the total) onto 24 chromosomes using a genetic map derived from six full-sib families and Hi-C contact data.

## Introduction

The sablefish (*Anoplopoma fimbria*) is a member of the teleost order Perciformes. While often called Alaskan black cod, they are more closely related to stickleback, sculpins, and lumpfish (suborder Cottioidei) than to cod (order Gadiformes) (Betancur-R *et al*. 2017; Maduna *et al*. 2022). Adult sablefish inhabit coastal regions of the eastern and western Pacific Ocean, from California to Japan (Sasaki 1985; Orlov and Biryukov 2005). Spawning occurs below 300 m (Mason *et al*. 1983). Embryos and larvae remain at depth for 1–2 months and arrive in the neustonic zone (ocean surface) as their yolk sacs are depleted (Mason *et al*. 1983; Deary *et al*. 2019). Juveniles move inshore and grow rapidly before returning to deep water (∼2 years) where they can live for more than 100 years (Mason *et al*. 1983).

Long-line hook and long-line pot traps are used to capture sablefish along the west coast of North America. Landings have shown a downward trend. The average annual commercial harvest over the 2012–2016 period was approximately half that of the 1992–1996 period (Hartley *et al*. 2020).

The short history of sablefish aquaculture was reviewed by Clarke and Pennell (2013) and Goetz *et al*. (2021). Currently, the only commercial producer is Golden Eagle Sablefish in British Columbia, Canada. Eggs and sperm from wild-caught and hatchery-reared captive broodstock are used to generate offspring that spend approximately 6 months in circulating saltwater tanks and up to 2 years in ocean pens. Mortality is high during the embryo and larval stages, and growth rate is highly variable, especially during the second year in ocean pens. In addition, a small percentage of sablefish develop spine curvatures during their second year. The percentage of curvatures and the magnitude of this dysmorphology vary among cohorts. Resistance to *Aeromonas salmonicida*, a bacterial pathogen that causes furunculosis, is another economically important trait that varies among sablefish individuals and cohorts (Vasquez *et al*. 2020).

Genomic data has played a major role in the improvement of long-domesticated species (e.g. dairy cattle) (Taylor *et al*. 2016) and in the development of new domestic lineages (e.g. Atlantic salmon) (Tsai *et al*. 2017). Improvements are achieved by identifying and using broodstock that carry genomic profiles associated with traits, such as rapid growth and pathogen resistance. Genomic tools may also be used to monitor and maintain genetic variation in domestic populations.

Prior to this work, Rondeau *et al*. (2013) sequenced the sablefish mitochondrial genome and identified microsatellite and SNP loci by sequencing expressed genes (EST and RNASeq data). These SNPs and microsatellites were used for genotyping two pedigrees and constructing a genetic map. Rondeau *et al*. (2013) also used genotype-by-sequencing (GBS) with k-mer analyses to identify markers associated with sex. In addition, two unpublished sablefish genome sequencing projects have been completed: BioProject PRJNA202249, assembly GCA_000499045.1, with 208506 contigs and contig N50 = 5156 bp (Illumina HiSeq), and BioProject PRJNA6567280, assembly GCA_000499045.2, with 50159 contigs and contig N50 = 29750 bp (Illumina HiSeq; PacBio; Illumina MiSeq). Here, we report the completion of a high-quality, chromosome-level sablefish genome. This genome assembly can be used to improve survival, growth rate, and pathogen resistance of sablefish in aquaculture. It also has the potential to strengthen the management of the wild sablefish fishery. While morphological data and mark-recapture data suggest that there are several wild populations, genetic analysis has not revealed any distinct population structure (e.g. Tsuyuki and Roberts 1969; Tripp-Valdez *et al*. 2012; Jasonowicz *et al*. 2016). Finally, sablefish aquaculture allows researchers access to individuals at all stages of development, making a diversity of studies along the continuum of discovery to applied research to commercialization possible in a species that is difficult to study in the wild. This genome assembly can enhance such studies.

## Methods

### Long-read (Oxford nanopore technology) genome sequencing

Heart and liver samples were removed from four freshly euthanized hatchery-reared individuals and flash-frozen. High molecular weight (HMW) DNA was extracted using the Nanobind Tissue Big DNA kit (Circulomics), following the standard dounce homogenizer HMW protocol (Handbook v.1.0). We then used the SRE-XS kit (Circulomics) according to the manufacturer's directions to decrease the presence of DNA fragments less than 10 Kbp long. Sequencing libraries were prepared using the ligation sequencing kits SQK-LSK109 and SQK-LSK110 for sequencing on R.9.4.1 flow-cells following the manufacturer's protocol. Raw sequence data was generated in FASTQ format using the Guppy Basecalling Software (v.3.4.3þf4fc735).

Nanopore reads from all four individuals were pooled and assembled using the Flye genome assembler program (v.2.9-b1774) (Kolmogorov *et al*. 2019) with default settings, except the genome-size was set to 0.8G and, to reduce assembly time, asm-coverage was set to 40. The estimated Nanopore read coverage was 77 × and the read N50 was 11031 bp, as estimated by the Flye genome assembly program. Subsequently, we used the software package Racon (v.1.4.16, parameters: -u) (Vaser *et al*. 2017) with Minimap2 (v.2.17-r941, parameters: -x map-ont) (Li 2018) to generate consensus sequences and polish the genome. To improve the quality, two additional rounds of polishing were performed using Pilon (v.1.22) (Walker *et al*. 2014) with default settings and the Illumina short reads from BioProject PRJNA869752 (139.4 Gbp or ∼213 × coverage). These reads were trimmed using Trimmomatic (v.0.39) (Bolger *et al*. 2014) with the following parameters: ILLUMINACLIP: TruSeq2-PE.fa: 2:30:10:2: keepBothReads, LEADING: 28, TRAILING: 28, MINLEN: 50, TOPHRED33, and then aligned to the Flye assembly using the bwa mem program with the parameter -M (Li 2013). Moreover, in August 2022, we submitted Illumina HiSeq data (774.2 million reads/139.4 Gbp) to NCBI (BioProject PRJNA869752).

### Genetic map

We produced a genetic map by resequencing the genomes of individuals from a microsatellite-based parentage study (Rubi *et al*. 2022). DNA was extracted using a Qiagen DNeasy Extraction Kit from fin clips from broodstock and progeny (N = 99). After DNA extraction, samples were sent to the Beijing Genomics Institute (BGI) for library preparation and sequencing. At BGI, short-insert libraries were prepared and sequenced (DNBseq, PE150). After sequencing, reads were filtered at BGI by removing adapter sequences, contamination, and low-quality reads. This was achieved using SOAPnuke (Chen *et al*. 2018) with the following parameters: -n 0.001 -l 10 - q 0.4 –adaMR 0.25 –ada_trim. The filtered reads were then aligned to the Flye-assembled long-read genome, described above, with the mitochondrion sequence from the assembly replaced with the NCBI reference mitochondrion sequence (accession NC_018119.1). The -M parameter was used in the bwa mem program (Li, 2013) (v.0.7.17). Read group information was added to the alignment files with Picard AddOrReplaceReadGroups (github.com/broadinstitute/picard) (v.2.26.3) and duplicate reads were marked using the MarkDuplicates command with the validation stringency set to lenient. Alignment files for each individual were then indexed using the SAMtools (Li *et al*. 2009) (v.1.12) index command.

Individual haplotypes were called using GATK v.3.8 (McKenna *et al*. 2010; DePristo *et al*. 2011; Van der Auwera *et al*. 2013) (HaplotypeCaller parameters: –genotyping_mode: DISCOVERY, –emitRefConfidence GVCF). All samples were then jointly genotyped using GATK GenotypeGVCFs. Genotypes were called on 10 Mbp intervals that were subsequently concatenated using the CatVariants command. BCFtools (Li 2011) and VCFtools (Danecek *et al*. 2011) were used to sort, compress, and index the GenotypeGVCFs output file. This VCF file was then filtered with VCFtools to remove indels and variants that did not pass several (population-level) genotyping criteria: variants with > 2 alleles, variants that were missing in ≥ 10% of the individuals, and those with a minor allele frequency < 0.05. This reduced the number of nucleotide variants (SNPs) from 21923895 to 6891741. To reexamine microsatellite-based familial relationships, relatedness was estimated using the vcftools –relatedness command (v. 0.1.15) (Danecek *et al.* 2011).

The genetic map was generated using LepMap3 (Rasta 2017). First, parental genotyping and error correction was performed using the ParentCall2 function (options: halfSibs = 1, remove NonInformative = 1). This was followed by marker filtering (dataTolerance = 0.001) using the Filtering2 command, which removes loci that lack variation or markers that show non-Mendelian inheritance (e.g. mitochondrial markers), leaving 6330039 loci. The logarithm of odds (LOD) score was then employed to identify linkage groups using the SeparateChromosomes2 function (options: sizeLimit = 50). Three iterations of the order markers command were used to order the markers that had been assigned to individual LGs by the LOD score-based SeparateChromosomes2 analysis. The ordered markers were then converted to the format used by Chromonomer (Catchen *et al*. 2020) using custom python scripts, and the marker sequences were aligned to the polished long-read genome assembly using bwa mem (with -M flag). The alignment was sorted with the SAMtools sort command. Chromonomer was then used to map scaffolds to their chromosome positions (−disable_splitting flag used). This placed roughly 95% of all nucleotides onto pseudo-chromosomes (620.9 Mbp were placed out of a genome length of 653.5 Mbp).

### Hi-C

For Hi-C sequencing, frozen sablefish heart tissue (stored at −70°C) was shipped to Canada's Michael Smith Genome Sciences Centre. A Hi-C library was generated using the Arima-HiC 2.0 kit (DocA160162 v00), which used the Swift Biosciences Accel-NGS S2S Plus DNA library and Indexing kits (Arima Genomics). The library was then sequenced on an Illumina NovaSeq (PE150). The Arima mapping pipeline (github.com/ArimaGenomics/mapping_pipeline) was used to align Hi-C reads to the genetic map-guided (i.e. Chromonomer) genome assembly, and to process the alignments. PhaseGenomics scripts (github.com/phasegenomics/juicebox_scripts) were used in combination with the AGP files from Chromonomer to generate a contact map (.hic file). Juicebox (v.1.11.08) (Durand *et al*. 2016) was used to visualize the contact map and make assembly corrections (e.g. ordering, orientation changes, and splitting misassembled contigs). PhaseGenomics scripts were then used to generate a new AGP file and sequence file. Sequences from the polished genome assembly were split based on the break report from PhaseGenomics using a custom python script.

### Comparative genomics

Genome assembly quality was assessed using two approaches. First, we used Benchmarking sets of Universal Single-Copy Orthologs (BUSCO) with the MetaEuk and Augustus gene predictors to determine how many core genes were included in the final sablefish assembly.

The core gene dataset used was actinopterygii_odb10 (Creation date: 2021-02-19, number of genomes: 26, number of single-copy orthologs: 3640). Second, the sablefish assembly was aligned to the genomes of the honeycomb rockfish (*Sebastes umbrosus*) (GCA_015220745.1), the threespine stickleback (*Gasterosteus aculeatus*) (GCA_016920845.1), and the lumpfish (*Cyclopterus lumpus*) (GCA_009769545.1) using D-Genies (Cabanettes and Klopp 2018). Low-complexity regions in the target genomes (e.g. repeats such as microsatellites and transposons) were masked by converting lower-case nucleotides to Ns. The Minimap2 (v 2.24) aligner was used within D-Genies with the repeatedness option set to few repeats. We also aligned the sablefish assembly to itself in search of large duplications. For this DGenies analysis, repeatedness was set to many repeats. Finally, we used Circos (Krzywinski *et al*. 2009) to visualize alignments of the threespine stickleback and sablefish genomes. These alignments were generated using BLASTn (megablast parameters: -outfmt 6, -max_hsps 40000) with stickleback chromosomes as query sequences. BLAST alignments were filtered with -min = 10000 (alignment length) and -gap = 100000 prior to Circos imaging. In addition to providing insight into the quality of the sablefish assembly, these genome alignments were used to investigate karyotype evolution among cottioids.

### Sex determination

The locations of the *gsdf*-associated insertions that Rondeau *et al*. (2013) determined were X chromosome and Y chromosome specific were investigated using BLASTn. We also searched for evidence of anti-Müllerian hormone (*amh*) gene duplication using the single copy sablefish *amh* gene (KC112919.1) as a query. Duplicated *amh* genes are sex-determining loci in other cottioids, including stickleback (Peichel *et al*. 2020), lumpfish (Holborn *et al*. 2021), and lingcod (*Ophiodon elongatus*) (Rondeau *et al*. 2016).

### Recombination landscape

The generation of a genetic map allowed us to compare recombination rates among chromosomes, within chromosomes, and between the same chromosomes inherited from males/sires vs females/dams. If recombination rate differs in maternally and paternally inherited chromosomes, a phenomenon called heterochiasmy, the recombination rate between the same linked markers will differ in the 2 sexes. As described above, LepMap3 Ordermarkers2 was used to calculate centimorgan (cM) positions for SNPs, and these data were compared to the physical distance between SNPs.

## Results and discussion

### Assembly

The new sablefish genome is 653.6 Mbp long, comprised of 8212 contigs (N50 = 2569385) and 7493 scaffolds (N50 = 26734982) (Table 1). By comparison, the genomes of two other cottioids, the threespine stickleback and the lumpfish, are 471.9 Mbp (PRJNA707557) and 572.9 Mbp (PRJNA625538) long, respectively.

**Table 1.** Sablefish genome sequencing and assembly results

Scaffolds	
Number of sequences	7493
N50	26734982
Contigs	
Number of sequences	8212
N50	2569385

Total sequence length 653543715.

The chromosome-level assembly was created using a genetic map and Hi-C sequence data. To generate the genetic map, we obtained short-read data from sablefish included in a microsatellite-based parentage analysis, seven broodstock individuals and 92 offspring (Rubi *et al*. 2022).

After genotyping and filtering, 6891741 SNP loci suitable for calculating pairwise relatedness were identified and from these data, 85 sablefish (six broodstock individuals and 79 offspring) were assigned to one of seven full-sib families (Supplementary Fig. 1). The three largest families (FAM1, FAM2, FAM3) included 24, 20, and 25 full-sib juveniles, respectively, and three smallest families (FAM5, FAM6, FAM7) had five or fewer offspring. FAM4 was excluded from further analyses because no parents were identified among the genotyped samples. Broodstock individual 8553 sired all offspring from FAM5 and FAM7, and individual 2794A was the dam of FAM6 and FAM7. Thus, there were full and half-sibs among the offspring in the three smallest families. With a LOD score threshold of 15, 691516 SNP loci could be assigned to one of 24 linkage groups. In total, 621 Mbp or 95% of the nucleotides were placed on the 24 linkage groups (with 5% on unplaced contigs/scaffolds). This essentially increased the scaffold N50 from 2.63 Mbp, which was the fragment N50 output from the Flye assembly program before corrections, to around 26 Mbp, as the Flye genome assembly program generally produces contigs only.

There were 9120165 intra 20Kbp Hi-C contacts identified after mapping Hi-C data to the contigs and scaffolds that had been sorted and ordered using the genetic map. Hi-C contact data exposed some misplaced and inverted contigs and scaffolds, which were corrected by hand using Juicebox (Durand *et al*. 2016). The scaffold N50 remained largely the same after using the Hi-C data, but this data was essential for corrections. We were able to remove roughly 10 Mbp of contigs that were misplaced in previous steps.

### Comparative genomics

The new sablefish genome contained almost all Actinopterygii core genes; BUSCO's default gene predictor, MetaEuk, detected 87.7% of the Actinopterygii core genes, and the Augustus gene predictor detected 97.8% (Supplementary Table 1). MetaEuk is translation-based: contigs and scaffolds are translated in all six reading frames, and the resulting amino acid strings are compared to those from a core-gene database, in our case, Actinopterygii_odb10. By contrast, Augustus, considers intron/exon boundaries (i.e. canonical splice sites) when identifying core-gene sequences. This time-consuming step appears to have made possible the discovery of more core-gene sequences than MetaEuk.

The D-Genies summary-of-identity estimate indicated, unexpectedly, that the sablefish genome is more similar to the lumpfish genome (81%) than the threespine stickleback genome (60%).

Sablefish (infraorder Anoplopomatales) is usually placed at the base of cottioid phylogenies (Rondeau *et al*. 2013; Betancur-R *et al*. 2017; Maduna *et al*. 2022) and was expected to be approximately equally dissimilar to threespine stickleback and lumpfish. Maduna *et al*. (2022) added a phylogenetic analysis to their study that incorporated fossil data, in particular, a 13 million-year-old stickleback. These analyses placed infraorder Gasterosteales (includes stickleback) as sister to the other cottioids, a hypothesis that is consistent with these summary-of-identity results. Each of the 24 chromosomes in sablefish was homologous to a single unique chromosome in honeycomb rockfish, the only non-cottioid in the comparison (Supplementary Fig. 2). This observation suggests that the sablefish genome structure is a good proxy for the ancestral cottioid genome.

**Fig. 1.**
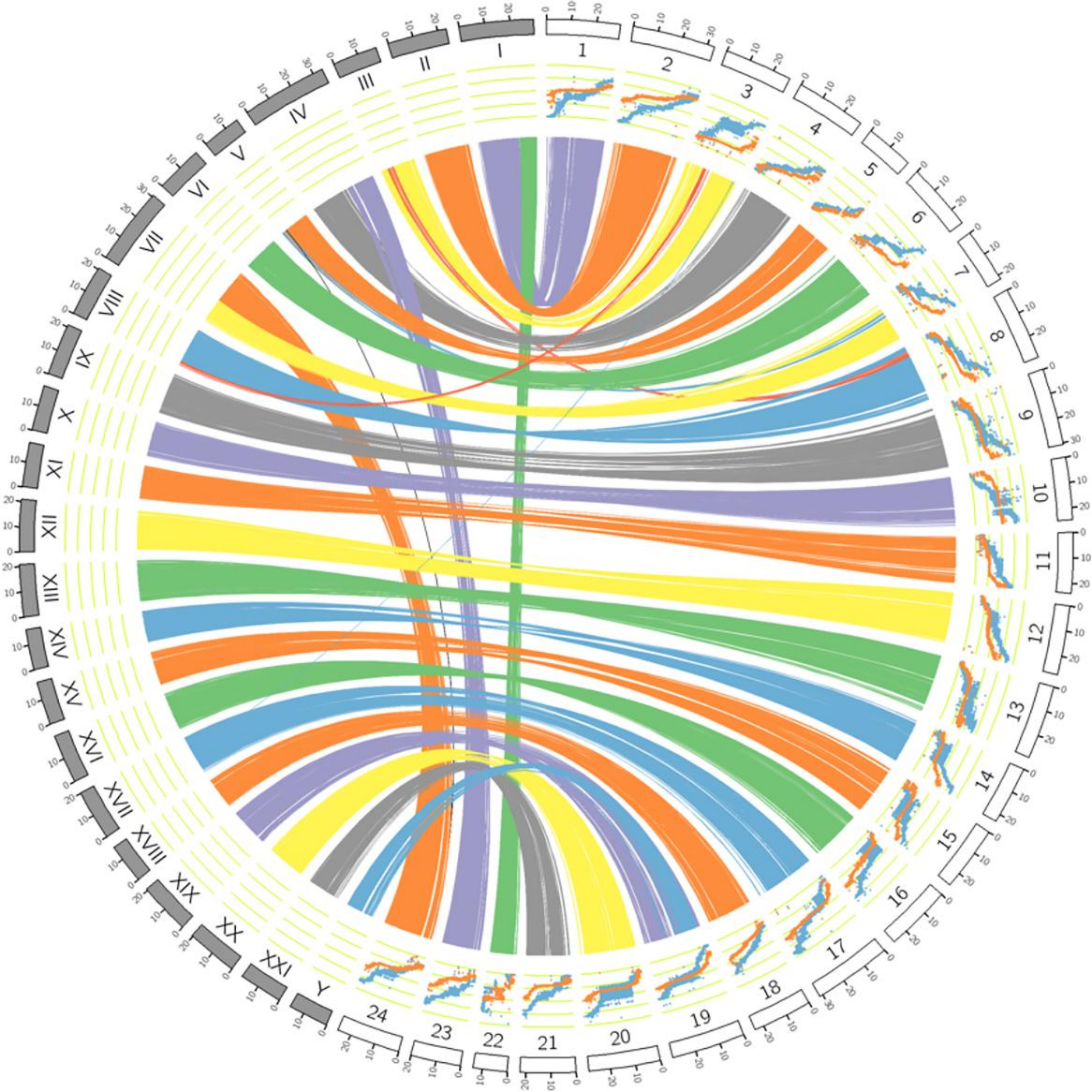
Threespine stickleback and sablefish Circos ribbon plot showing chromosome-level alignments. Threespine stickleback has 21 chromosomes, 19 and Y are homologs, but displayed as distinct chromosomes. Sablefish have 24 chromosomes. Threespine stickleback fusion chromosomes 1, 4, and 7 align to sablefish chromosomes 1 + 22, 4 + 23 and 7 + 24, respectively. A sablefish-specific, ∼2 Mbp, translocation (chromosome 3 to chromosome 8) is also shown. Chromosome-level alignments/dot plots (Supplementary Figs. 2–4) show that this translocation is derived in sablefish. This figure also shows the relationship between chromosome length in cM (X-axis) and length in base pairs (y-axis) for each sex. Each dot on the figure represents a genetic marker with the female cM position (blue) and male cM position (orange) on the Y-axis, and the physical position on the X-axis.

Within Cottioidei, chromosome numbers vary and this complicates synteny based naming. Rondeau *et al*. (2013) used the threespine stickleback genome as a guide when they labeled the 24 sablefish linkage groups, and we have followed that scheme: Eighteen of the new sablefish chromosome sequences aligned to a distinct threespine stickleback chromosome (1:1 alignments) and they were given the name/ID of the threespine stickleback homolog. In this study, and in Rondeau *et al*. (2013), there were three 2:1 alignments: sablefish chromosomes 1 + 22 align to distinct regions of threespine stickleback chromosome 1, sablefish chromosomes 4 + 23 align to threespine stickleback chromosome 4, and sablefish chromosomes 7 + 24 align to threespine stickleback chromosome 7 (Fig. 1; Supplementary Fig. 3). Liu *et al*. (2022) recently produced chromosome-level genome sequences for close relatives of the threespine stickleback, the ninespine stickleback (*Pungitius pungitius*) with 21 chromosomes, and the fourspine stickleback (*Apletes quadracus*) with 23 chromosomes. Their naming system was not developed in the context of an ancestral 24-chromosome karyotype; Liu *et al*. (2022) described threespine stickleback chromosomes 4 and 7 as fusions of fourspine stickleback 4 + 22 and 7 + 23 respectively.

While chromosome fusion events explain the 2:1 alignments in stickleback (Liu *et al*. 2022), our analyses provided a new perspective on the origin of lumpfish chromosome 25 (Holborn *et al*. 2021); it is possibly a chimera of large fragments originally present on lumpfish chromosomes 13 (NC_046978.1) and 16 (NC_046981.1) (Supplementary Fig. 4). The interspecific chromosome-level alignments (all three) also exposed a sablefish-specific, approximately 2 Mbp, translocation (Fig. 1). The intraspecific alignment (Supplementary Fig. 5) shows that this was not a case of duplicative transposition. Last, the high degree of synteny among the species surveyed indicates that QTL mapping in one species will help focus searches for candidate loci in others.

### Sex determining locus

By surveying GBS sequence data for sex-specific k-mers (31-mers), Rondeau *et al*. (2013) uncovered an insertion upstream of *gsdf* (*gonadal soma derived factor*) in sablefish that was only present in males. Males have insertion-heterozygote genotypes at this locus and Rondeau *et al*. (2013) proposed, based on observations from *Oryzias luzonensis* (Myosho *et al*. 2012), that the insertion influenced *gsdf* expression in a manner that determines sex. It is now clear that the TE-derived insert upstream of *gsdfY* (the male-specific allele) has far more binding sites for transcription factors, Dmrt1 and Wt1(-KTS), than the X-specific allele, *gsdfX*, and that this difference drives *gsdf* expression much earlier in development in males compared to females (Herpin *et al*. 2021). We used BLAST to locate *gsdf* in the chromosome-level assembly and confirmed its position on chromosome 14.

In *O. luzonensi,* the male-specific *gsdf* allele replaced *dmy* as the sex-determining locus (Myosho *et al*. 2012). In sablefish, sex determination via *gsdfY/gsdfX* allelic divergence has evolved in a lineage (Cottioidei) where an *amh* driven sex-determination system is most common. We used BLAST to locate the sablefish *amh* gene. In lumpfish (Holborn *et al*. 2021), stickleback (Peichel *et al*. 2020) and lingcod (Rondeau *et al*. 2016), and indeed in many non-cottioids (e.g. Hattori *et al*. 2012; Li *et al*. 2015; Song *et al*. 2021), duplicated *amh* genes have evolved sex-determination roles. We found only one *amh* gene in sablefish and phylogenetic analyses (not shown) indicated that this single copy gene was the pro-ortholog of paralogs, in stickleback, lumpfish and lingcod, which were all derived from species-specific duplication events.

### Recombination landscape

The output from Lep-Map3 (genetic map) indicated 2–3 recombination events per chromosome per offspring individual (N = 83). The recombination rate varied among chromosomes (Supplementary Fig. 6) but was not correlated with chromosome length. Recombination rates were also higher among maternal chromosomes (except chromosome 22) than in paternally-derived chromosomes (Supplementary Fig. 6). In addition, recombination rate varies along the lengths of chromosomes, as shown in MareyMaps (Siberchicot *et al*. 2017) included in Fig. 1.

### Future

The sablefish genome assembly reported here can form the foundation for SNP-based surveys (i.e. SNP panels and/or skim-sequencing-based genotyping) designed to detect genetic markers associated with survival, growth rate, and pathogen resistance. Beyond its contribution to sablefish domestication, the genome assembly can be a useful resource for the management of the wild sablefish fishery.

## Supplementary Material

jkad089_Supplementary_Data

## Data Availability

The chromosome-level sablefish (*Anoplopoma fimbria*) genome assembly described here was submitted to NCBI under project number PRJNA869752. The NCBI-annotated genome can be explored and analyzed at https://www.ncbi.nlm.nih.gov/genome/gdv? org=anoplopoma-fimbria&group=anoplopoma. Supplemental material available at G3 online.
