# Supplementary material for: Sablefish (Anoplopoma fimbria) chromosome-level genome assembly
Source: G3 (Bethesda). 2023 Apr 25;13(7):jkad089. doi: 10.1093/g3journal/jkad089 (PMC10320756; doi:10.1093/g3journal/jkad089)
Supplement: jkad089_Supplementary_Data [file jkad089_supplementary_data.zip › Figure_S1_G3-2022-404015.docx]

Fam3Fam2Fam6Fam1Fam7Fam4 (excluded from pedigree file)Fam5Fam3Fam2Fam6Fam1Fam7Fam4 (excluded from pedigree file)Fam5
