# Supplementary material for: Sablefish (Anoplopoma fimbria) chromosome-level genome assembly
Source: G3 (Bethesda). 2023 Apr 25;13(7):jkad089. doi: 10.1093/g3journal/jkad089 (PMC10320756; doi:10.1093/g3journal/jkad089)
Supplement: jkad089_Supplementary_Data [file jkad089_supplementary_data.zip › Supplemental Figure Legends.docx]

Figure S1. Relatedness data for 99 sablefish included in a microsatellite-based parentage study (Rubi *et al*. 2022). Eighty-five fish were sorted into one of seven full-sib families (FAM1 - FAM7) based on relatedness estimated from 6891741 SNP loci. Six families (FAM1, FAM2, FAM3, FAM5, FAM6, and FAM7) were used to generate a pedigree file for genetic map reconstruction.

Figure S2. Sablefish (DGenies query) and honeycomb rockfish (DGenies target) chromosome alignments. Chromosome order based upon rockfish chromosomes (1-24, top left to top right). Arrow shows ~2 Mbp of sablefish chromosome 3 sequence aligned to rockfish chromosome 5, which is homologous to sablefish chromosome 8.

Figure S3. Sablefish (DGenies query) and threespine stickleback (DGenies target) chromosome alignments ordered by stickleback chromosomes (start from 1 at top left). Sablefish chromosomes have the same names/numbers as threespine stickleback homologs, with three exceptions; stickleback chromosome 1 is homologous to sablefish chromosomes 1+22, stickleback chromosome 4 is homologous to sablefish chromosomes 4+23, and stickleback chromosome 7 is homologous to sablefish chromosomes 7+24. Arrow shows ~2Mbp region of sablefish chromosome 3 aligned to stickleback chromosome 8, which is homologous to sablefish chromosome 8.

Figure S4. Sablefish (DGenies query) and lumpfish (DGenies target) chromosome alignments. Chromosome ordered based upon the lumpfish chromosomes, 1-25, from top left to right. Arrow shows ~2Mbp region of sablefish chromosome 3 aligned to lumpfish chromosome 4 (which is the homolog of sablefish chromosome 8). In addition, lumpfish chromosome 25 is comprised of a sequence that is homologous to regions of sablefish chromosomes 1 and 20, suggesting that this ‘extra’ chromosome is a fusion of fragments originally from lumpfish chromosomes 13 and 16.

Figure S5. Sablefish (DGenies query) and sablefish (DGenies target) chromosome alignments. This analysis shows no evidence of large-scale duplication events within the sablefish genome.

Figure S6. Recombination rates (average cM distance/physical distance in Mbp) for chromosomes in offspring derived from the dam/female (blue) and sire/male (orange) in 83 (F1) offspring.
