# Supplementary material for: Sablefish (Anoplopoma fimbria) chromosome-level genome assembly
Source: G3 (Bethesda). 2023 Apr 25;13(7):jkad089. doi: 10.1093/g3journal/jkad089 (PMC10320756; doi:10.1093/g3journal/jkad089)
Supplement: jkad089_Supplementary_Data [file jkad089_supplementary_data.zip › Table S1.docx]

Table S1. BUSCO (v.5.3.2) analysis of the sablefish (*A. fimbria*) genome assembly using the MetaEuk and Augustus gene-prediction tools. The core gene dataset was *actinopterygii_odb10*.

|  | MetaEuk | Augustus |
| --- | --- | --- |
| Complete BUSCOs | 3193 (87.7%) | 3560 (97.8%) |
| Complete and single-copy BUSCOs | 3157 (86.7%) | 3516 (96.6%) |
| Complete and duplicated BUSCOs | 36 (1.0%) | 44 (1.2%) |
| Fragmented BUSCOs | 215 (5.9%) | 12 (0.3%) |
| Missing BUSCOs | 232 (6.4%) | 68 (1.9%) |
| Total BUSCO groups searched | 3640 | 3640 |
|  |  |  |
