# Supplementary figures and images for: Sablefish (Anoplopoma fimbria) chromosome-level genome assembly
Source: G3 (Bethesda). 2023 Apr 25;13(7):jkad089. doi: 10.1093/g3journal/jkad089 (PMC10320756; doi:10.1093/g3journal/jkad089)

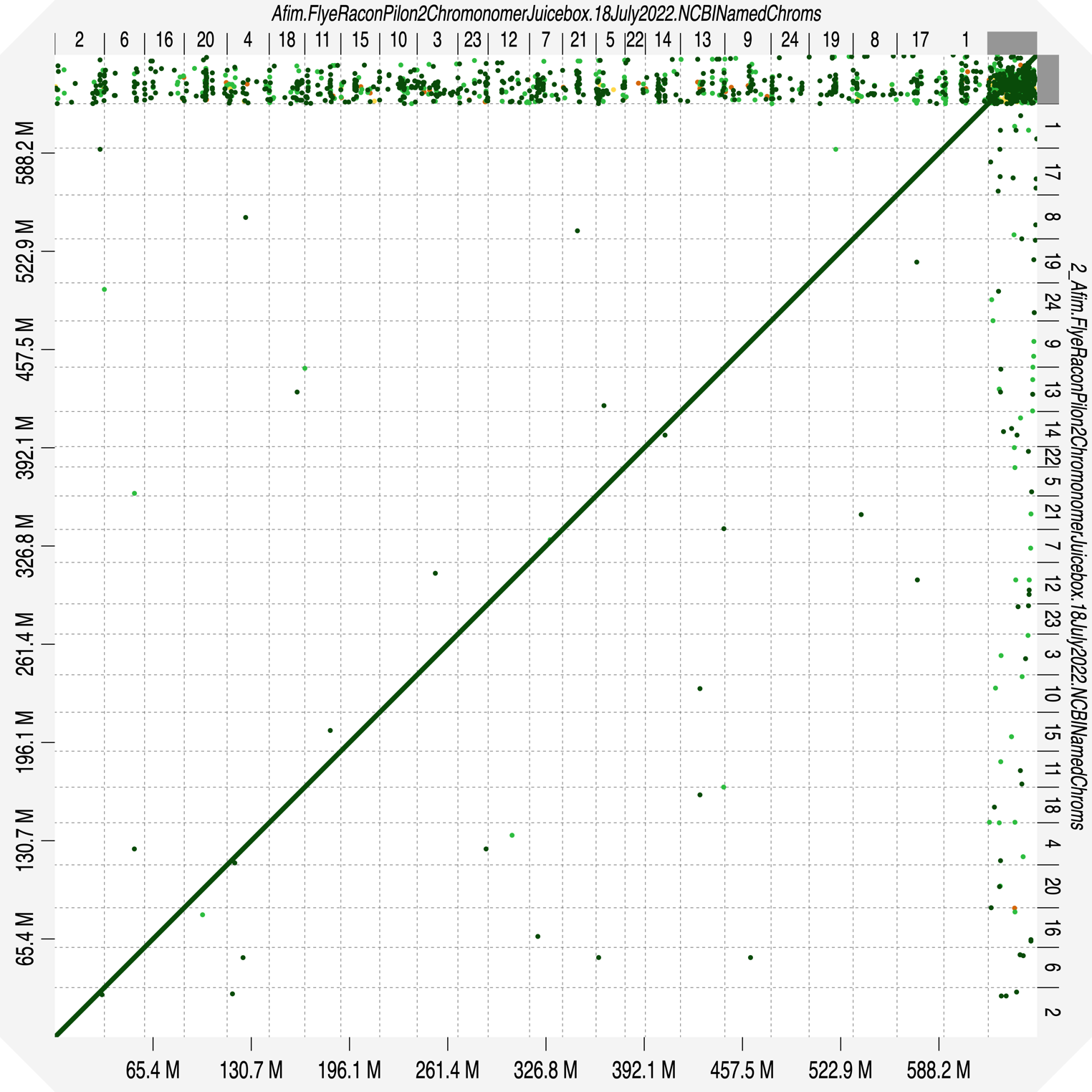

Supplement: jkad089_Supplementary_Data [file jkad089_supplementary_data.zip › Figure_S5_G3-2022-404015.docx]

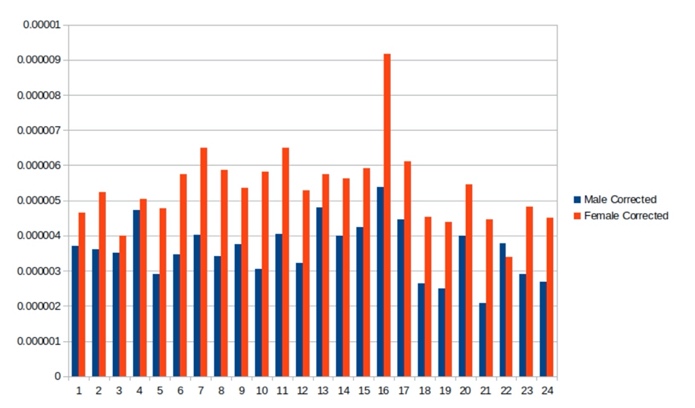

Supplement: jkad089_Supplementary_Data [file jkad089_supplementary_data.zip › Figure_S6_G3-2022-404015.docx]
